# The cold atmospheric pressure plasma-generated species superoxide, singlet oxygen and atomic oxygen activate the molecular chaperone Hsp33

**DOI:** 10.1098/rsif.2023.0300

**Published:** 2023-10-25

**Authors:** Tim Dirks, Marco Krewing, Katharina Vogel, Julia E. Bandow

**Affiliations:** Applied Microbiology, Faculty of Biology and Biotechnology, Ruhr University Bochum, Bochum, Germany

**Keywords:** dielectric barrier discharge, plasma resistance, plasma protein interaction, reactive oxygen species, aggregation, zinc coordination

## Abstract

Cold atmospheric pressure plasmas are used for surface decontamination or disinfection, e.g. in clinical settings. Protein aggregation has been shown to significantly contribute to the antibacterial mechanisms of plasma. To investigate the potential role of the redox-activated zinc-binding chaperone Hsp33 in preventing protein aggregation and thus mediating plasma resistance, we compared the plasma sensitivity of wild-type *E. coli* to that of an *hslO* deletion mutant lacking Hsp33 as well as an over-producing strain. Over-production of Hsp33 increased plasma survival rates above wild-type levels. Hsp33 was previously shown to be activated by plasma *in vitro*. For the PlasmaDerm source applied in dermatology, reversible activation of Hsp33 was confirmed. Thiol oxidation and Hsp33 unfolding, both crucial for Hsp33 activation, occurred during plasma treatment. After prolonged plasma exposure, however, unspecific protein oxidation was detected, the ability of Hsp33 to bind zinc ions was decreased without direct modifications of the zinc-binding motif, and the protein was inactivated. To identify chemical species of potential relevance for plasma-induced Hsp33 activation, reactive oxygen species were tested for their ability to activate Hsp33 *in vitro*. Superoxide, singlet oxygen and potentially atomic oxygen activate Hsp33, while no evidence was found for activation by ozone, peroxynitrite or hydroxyl radicals.

## 1. Introduction

Cold atmospheric pressure plasmas have an emerging application range in medicine and biology, especially for sterilization or decontamination of materials [1], since it is known that plasma inactivates bacteria [2] and can remove proteins and DNA, respectively [3,4]. Despite the widespread medical and biological application, the underlying molecular mechanisms of bacterial inactivation are still not completely elucidated. Proteins are the most abundant biomolecules inside a cell, and thus a lot of investigations have been carried out to obtain information about the effects of plasma on proteins *in vivo*. In most studies the inactivation of key metabolic proteins was observed [5,6]. Furthermore, it has previously been shown that protein aggregation occurs during plasma treatment in yeast cells [7]. This is based on chemical modifications of amino acids. The function of a respective amino acid with regard to enzyme activity determines whether, for example, inactivation takes place due to a loss of catalytic function [6] or due to a change in protein fold that leads to reduced enzyme activity and potentially aggregation [8–10]. Sulfur-containing amino acids (methionine and cysteine) are especially susceptible to plasma-induced modifications, followed by aromatic amino acids (tryptophan, phenylalanine, tyrosine, histidine) [8]. Besides protein modification and unfolding, degradation of proteins is another observed effect upon plasma treatment [5,11]. The exact mechanism of protein degradation is still under investigation; however, it was confirmed that proteins are degraded to peptides of different sizes due to cleavage of peptide bonds, resulting in the formation of new terminal amino functions [12]. In contrast to plasma-induced inactivation of most proteins, the molecular chaperone heat shock protein 33 (Hsp33) of *Escherichia coli*, which is encoded by the gene *hslO* [13], is activated by plasma [14]. Here, oxidation of regulatory cysteine residues and a distinct unfolding trigger the activation process. Chaperones in general support other proteins in folding correctly or in preventing aggregation of unfolded proteins. Hsp33 acts ATP-independently as a holdase and binds unfolding proteins, thus preventing their aggregation [15]. Hsp33 consists of three main domains: a core domain, which contains a substrate binding site, a linker, and a redox sensing region [16,17]. Of the six cysteine residues of Hsp33, four are highly conserved and play a role in its activation. These four cysteines are part of the redox switch domain and coordinate a zinc ion [18]. In the protein's inactive state and under non-oxidizing conditions, the linker region masks the substrate binding site, thus preventing binding of unfolded proteins [19,20]. It was shown that a single mutation destabilizing the linker region renders the protein constitutively active, demonstrating the importance of the linker region in Hsp33 activation [20]. Oxidation with a slow-acting oxidant (e.g. H_2_O_2_) leads to the formation of one disulfide bond and a slight detachment of the linker region from the substrate binding site. For a complete protein activation, further unfolding due to elevated temperature (43°C) or exposure to a fast-acting and protein-unfolding oxidant (e.g. HOCl) is necessary [20–22]. Oxidation of all four regulatory cysteine residues of Hsp33 leads to formation of two disulfide bonds, the release of the zinc ion, and a distinct unfolding, rendering the protein fully active [15,18,22,23]. Oxidation and unfolding together cause the exposure of hydrophobic residues of the substrate binding site that interact with the hydrophobic regions of unfolded proteins [24]. Reducing conditions, such as thioredoxin *in vivo* or dithiothreitol (DTT) *in vitro*, and supply of zinc ions lead to an inactivation of Hsp33 due to reduction of the disulfide bonds, binding of a zinc ion to yield the thiolate state of the cysteine residues, and re-folding [25]. In a previous study it has been shown that Hsp33 is also activated by plasma treatment using the Soft Bond DBD designed for fingernail treatment [14], highlighting its unusual plasma–protein interaction profile.

Here, using the clinically relevant plasma device PlasmaDerm (Cinogy, Duderstadt, Germany) designed for dermatological applications, we investigated the potential for bacterial plasma resistance development mediated by Hsp33 over-production *in vivo*. We further investigated the mechanisms underlying plasma-induced Hsp33 activation *in vitro*.

## 2. Experimental procedures

### 2.1. Plasma sources

If not indicated otherwise, plasma treatment was carried out using the PlasmaDerm dielectric barrier discharge (DBD) (Cinogy, Duderstadt, Germany) with an electrode diameter of 20 mm. Plasma was ignited in ambient air using *V*_RMS_ = 13.5 kV and a trigger frequency of 300 Hz. Samples (40 µl) were placed on top of a metal plate on a grounded counter electrode with a 1 mm distance to the driven electrode.

In addition, a microscale atmospheric pressure plasma jet (µAPPJ) [26] was used. Helium (1.4 slm, 5.0 purity) with varying oxygen admixtures (4.8 purity) was used as feed gas. Plasma ignition was driven with 13.56 MHz and 230 *V*_RMS_. The distance of the sample to the nozzle of the jet was varied from 0 to 20 mm.

### 2.2. Plasma sensitivity assay

To investigate the plasma resistance of different *E. coli* strains (BW25113, BW25113 Δ*hslO*, BW25113 Δ*hslO* + pCA24N::*hslO*), a colony forming unit (CFU)-based assay was conducted. To this end, 5 ml of LB medium were inoculated with 50 µl of a stationary-phase preculture and incubated shaking at 37°C. For strains carrying a plasmid for protein over-production, chloramphenicol and different amounts of isopropyl β-d-1-thiogalactopyranoside (IPTG) were added directly at inoculation of the main culture. At OD_600_ = 0.3, the cultures were first diluted with LB medium to OD_600_ = 0.01 and subsequently 1 : 500. Of this low-density cell suspension, 40 µl were treated with plasma. Samples were placed as droplets on top of in-house built metal plates, which are formed in such a way that they can be placed onto reaction tubes in place of the lid. This allowed for complete recovery of the samples by centrifugation. After plasma treatment, samples were plated onto LB agar for colony counting next day. Untreated controls were generated by exposing 40 µl of the cell suspension to feed gas only, without plasma ignition. CFU after plasma treatment were set in relation to CFU of untreated controls.

### 2.3. SDS-PAGE and western blot

For detection and quantitative analysis of over-produced proteins, SDS-PAGE and western blot analysis according to standard protocols were performed [27]. At an OD_600_ of 0.3, cells (1 ml) from the undiluted sample originating from the DBD sensitivity assay were pelleted, resuspended in 75 µl denaturing loading buffer, and boiled at 95°C for 10 min. After removal of cell debris by centrifugation (13 000 rpm, 10 min), 5 µl were analysed by SDS-PAGE. Detection of the His_6_-tagged proteins, which were over-expressed using plasmids from the ASKA collection [28], was carried out using the Penta-His antibody (Qiagen, Hilden, Germany). Relative signal intensities were determined using the Image Studio software (LI-COR, Nebraska, USA).

### 2.4. Hsp33 preparation

Purification of wild-type untagged Hsp33 was performed as described elsewhere with minor modifications [15]. In brief, a high-density cell culture of *E. coli* BB7222 containing the plasmid pBAD30::*hslO* was induced using 0.2% arabinose for 5 h. After cell harvest and sonication, cell free lysate was used to perform anion exchange chromatography, followed by two consecutive size exclusion chromatography runs (Superdex 75 and Superdex 200, GE Healthcare, USA). Hsp33 was eluted in potassium phosphate buffer (40 mM, pH 7.5). Afterwards, the protein was concentrated using centrifugal filter units (cut-off 10 kDa), aliquoted and stored at −20°C.

Prior to any experiment employing Hsp33, the protein was reduced (Hsp33_red_) by adding 5 mM DTT and 115 µM ZnCl_2_ to 5 mg ml^−1^ Hsp33. This was followed by incubation at 37°C for 2 h [23]. Chemically oxidized Hsp33 (Hsp33_HOCl_) was used as a positive control. To generate it, sodium hypochlorite (Sigma-Aldrich, St Louis, MO, USA) was diluted in water yielding an equimolar mixture of hypochlorous acid (HOCl) and its conjugate base (OCl^−^). Hsp33_red_ was oxidized by incubation with a 50-fold molar excess of HOCl at 30°C and 300 rpm for 10 min. After reduction or oxidation, Hsp33_red_ and Hsp33_HOCl_ were purified using the Micro Bio-Spin Columns-P30 (Bio-Rad, Munich, Germany) according to manufacturers’ instructions.

To test if oxidation of Hsp33 (chemically or by plasma) was reversible, the oxidized protein was diluted to 5 µM with potassium phosphate buffer. Afterwards, 5 mM DTT and 5 µM ZnCl_2_ were added. This reaction mixture was incubated at 30°C for 20 h [22].

Protein concentrations were determined using Bradford reagent according to standard protocols [29].

### 2.5. Quantitation of amino groups

Amino acids and polypeptides with amino termini were detected using ninhydrin as described elsewhere [12,30]. In short, 100 µl protein samples were mixed with 200 µl ninhydrin solution containing 2% (w/v) ninhydrin, 75% (v/v) ethanol, 1 M acetate buffer (pH 5.5) and 0.25% (w/v) SnCl_2_. Ninhydrin reacts with amino groups and forms the so-called Ruheman's purple [30]. The reaction mixture was incubated at 100°C for 10 min. After centrifugation at 13 000 rpm for 5 min, the absorption of the supernatant was measured at OD_575_ using a plate reader (µQuant, Bio-Tek Instruments, Vermont, USA). Acidic hydrolysis was performed as a control. To this end, 10 µl Hsp33 (1 mg ml^−1^) were incubated with 10 µl hydrochloric acid (37%) at 100°C for at least 24 h and then measured using ninhydrin.

### 2.6. Chaperone activity assay

Hsp33 chaperone activity was determined as described by Ilbert *et al*. [21]. The substrate for the activity assay was *E. coli* citrate synthase GltA, which was over-expressed using the strain BL21 pCA24N::*gltA* [28]. The His-tagged citrate synthase was purified using a nickel-NTA column according to standard protocols [27]. Purified citrate synthase (12 µM) was denatured chemically by incubation with 4.5 M guanidinium chloride in 40 mM HEPES (pH 7.5) overnight at room temperature. The chaperone activity assay was performed in a quartz cuvette by means of measuring light scattering. To 900 nM Hsp33 in 40 mM HEPES (1.6 ml final volume), 150 nM denatured citrate synthase was added. The reaction mixture was incubated at room temperature for 5 min while stirring at 800 rpm. Light scattering caused by aggregating citrate synthase was measured using a fluorescence spectrometer (FP-8500, Jasco, Gross-Umstadt, Germany). Light scattering of samples containing Hsp33_red_ was set to 0% chaperone activity, while light scattering with Hsp33_HOCl_ was set to 100% chaperone activity. Relative chaperone activities of plasma-treated Hsp33 samples were calculated using the range given by the positive and negative control and assuming an inverse linear relationship between light scattering and chaperone activity.

For experiments involving scavengers, Hsp33 (1 mg ml^−1^) was treated in the presence of 0.1 mM, 1 mM or 10 mM L-histidine and/or Mn(III)tetrakis(4-benzoic acid)porphyrin chloride (MnTBAP) in a total volume of 40 µl for 120 s.

### 2.7. Quantitation of free thiols

For analysis of the cysteine oxidation states of Hsp33, the DTNB assay as described by Ellman was used [31]. Hsp33 was diluted to 5 µM in potassium phosphate buffer (40 mM, pH 7.5) containing 6 M guanidinium chloride. 1 mM 5,5′-dithiobis-(2-nitrobenzoic acid) (DTNB) was added. The reaction mixture was incubated at room temperature in the dark for 15 min. Afterwards, absorption at 412 nm was measured. The number of thiols per protein was calculated by application of the Beer–Lambert law (*ε*_412_ = 13 880 M^−1^ cm^−1^).

### 2.8. Far-UV circular dichroism spectroscopy

Far-UV circular dichroism (CD) spectroscopy was carried out at room temperature with a spectropolarimeter (J-815, Jasco, Gross-Umstadt, Germany) using a quartz cuvette with 1 mm path length. Hsp33 was diluted to 0.15 mg ml^−1^. The spectra were buffer corrected and converted to molar ellipticity [32]. For display of the results, mean values of triplicates were calculated and the resulting curves were smoothed using the MATLAB software (R2018) using a smoothing span of 5%.

### 2.9. Detection of protein carbonylation

The carbonylation of plasma-treated Hsp33 was investigated following a protocol described by Levine *et al*. [33]. 40 µl protein (1 mg ml^−1^) were mixed with 40 µl 2,4-dinitrophenylhydrazine (DNPH). After incubation in the dark for 10 min, 20 µl NaOH (6 M) were added followed by a second incubation in the dark for 10 min. Untreated Hsp33 served as negative control. Absorption at 405 nm was detected using a plate reader (µQuant, Bio-Tek Instruments, Vermont, USA).

### 2.10. Determination of zinc binding by Hsp33

The zinc-binding ability of plasma-treated Hsp33 was determined by two methods, first by tracking structural changes by measuring intrinsic tryptophan fluorescence of the protein and second by application of the zinc-sensitive dye zincon and absorption measurements.

Hsp33 was prepared as described before. After plasma treatment, the protein was diluted to 5 µM in Tris buffer (10 mM, pH 7.5) and reduced overnight with 5 mM DTT at 30°C without addition of ZnCl_2_. Next day, different amounts of ZnCl_2_ were added to aliquots of the reduced Hsp33 and the reaction mixtures were incubated at 37°C for 90 min to allow for zinc ion incorporation. The zinc-induced conformational changes were monitored on the basis of changes in tryptophan fluorescence (*λ*_ex_ = 280 nm; *λ*_em_ = 340 nm). The measurements were carried out using a fluorescence spectrometer (FP-8500, Jasco, Gross-Umstadt, Germany).

Alternatively, zinc-binding ability of Hsp33 was determined using zincon according to Macnair & Smirnoff [34]. Oxidized Hsp33 (chemically or by plasma) was reduced overnight without zinc addition. Afterwards 1 mM zinc was added to 50 µM Hsp33 and incubation took place at 37°C for 90 min. The remaining unbound zinc was removed using centrifugal filter units (cut-off 10 kDa) by washing five times. The Hsp33-bound zinc was released by addition of a 50-fold molar excess of H_2_O_2_ and incubation at 65°C for 1 h. Zinc was quantified mixing 0.003% zincon (dissolved in water) with 25 µM Hsp33. After 15 min of incubation, absorption at 606 nm was measured. Two proteins that do not bind zinc, RidA_Ec_ and CnoX_Ec_, served as controls for unspecific zinc binding. To calculate the number of zinc ions bound per Hsp33 molecule, the amount of zinc bound by the controls (RidA_Ec_ and CnoX_Ec_) was subtracted from the Hsp33 measurements.

### 2.11. Treatment of Hsp33 with reactive oxygen species

The activation of Hsp33 by different reactive oxygen species (ROS) (supplied as described in the following) was analysed by incubating 0.5 mg ml^−1^ protein in a volume of 100 µl at room temperature or 43°C together with the respective ROS or ROS producer. Afterwards, activity of Hsp33 was determined using the citrate synthase assay.

### 2.12. Superoxide

Generation of superoxide $\left(O_{2}^{-}\right)$ was performed according to Nishikimi *et al*. and Ponti *et al*. [35,36]. The reaction mixture consisted of varying NADH amounts, 5 µM phenazine methosulfate (PMS), 25 µM nitro blue tetrazolium (NBT) and Hsp33. PMS catalyses the reduction of molecular oxygen to superoxide by oxidation of NADH to NAD^+^. The decrease of NADH was monitored at 340 nm. As control, NBT was used, which also reacts with superoxide, forming formazan which absorbs at 560 nm (electronic supplementary material, figure S1).

### 2.13. Peroxynitrite

Peroxynitrite was synthesized according to Uppu & Pryor using isopentyl nitrite and hydrogen peroxide [37].

### 2.14. Singlet oxygen

Singlet oxygen was generated using methylene blue according to Lamberts & Neckers and Kochevar & Redmond [38,39]. Hsp33 was incubated in a volume of 100 µl together with 1 µl of a 1 M methylene blue solution (dissolved in ethanol). Methylene blue was excited at 650 nm for different durations to yield different amounts of singlet oxygen. Excitation at 650 nm was carried out using a UV–visible/NIR spectrophotometer (V-750, Jasco, Gross-Umstadt, Germany).

### 2.15. Hydroxyl radicals

The Fenton reaction was used to generate •OH. 0.5 mM FeSO_4_ was incubated together with 10 mM H_2_O_2_ and Hsp33 for 1 h [40]. Formation of •OH was monitored using terephthalic acid (1 mM in potassium phosphate buffer), which reacts with •OH to 2-hydroxyterephthalate (HTA) resulting in a fluorescence signal (*λ*_ex_ = 315 nm; *λ*_em_ = 425 nm) [41]. Impact of •OH radicals was not tested at 43°C, since H_2_O_2_ already activates Hsp33 at elevated temperatures.

### 2.16. Atomic oxygen

The µAPPJ was used for generation of atomic oxygen. To this end, 200 µl of a 1 mg ml^−1^ Hsp33 solution were treated for 30 s with the effluent of the plasma jet, while the oxygen content was varied (0–1% O_2_). This variation of oxygen admixture leads to production of different amounts of atomic oxygen.

## 3. Results

### 3.1. Over-production of Hsp33 increases plasma resistance

**Figure 1.**
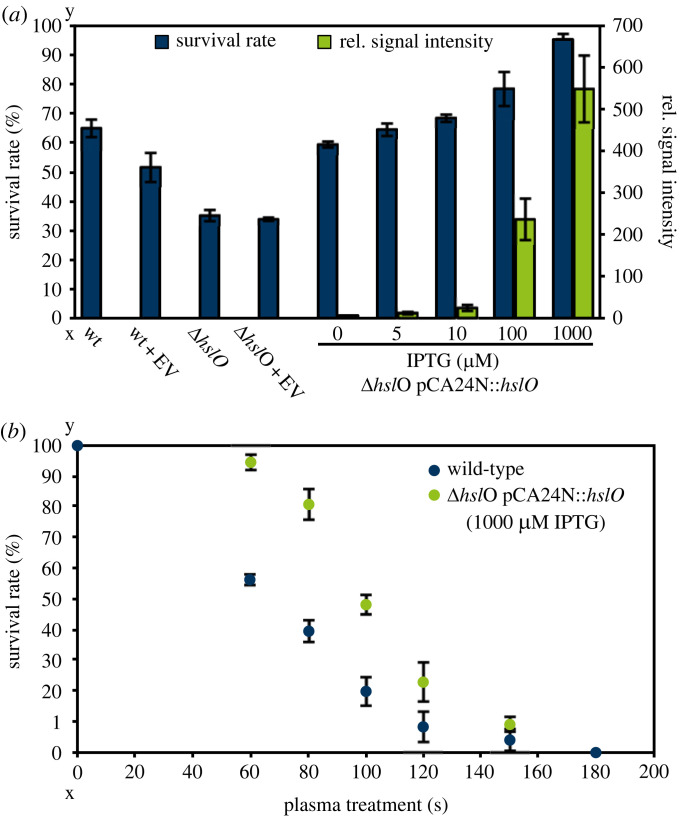
Relevance of *hslO* for plasma resistance. (*a*) Survival rates (blue) after 60 s plasma treatment of *E. coli* wild-type BW25113, the deletion strain Δ*hslO* (both with or without the empty vector pCA24N) and the complemented strain Δ*hslO* + pCA24N::*hslO* induced with different IPTG concentrations. Relative signal intensities monitored in western blots for the IPTG-induced complemented strains are displayed in green. (*b*) Survival of *E. coli* wild-type (blue) and the complemented strain Δ*hslO* + pCA24N::*hslO* induced with 1000 µM IPTG (green) at different plasma treatment times. (*a,b*) Survival of the untreated control was set to 100% survival rate. Means and standard deviations representing three experiments are shown. wt: wild-type; EV: empty vector pCA24N.

Proteins are the most abundant biomolecules in cells and it was independently shown that proteins quickly unfold and get inactivated by plasma treatment [5,6,9]. Thus, we hypothesized that protection of proteins from the effects of plasma leads to increased plasma resistance. While various definitions of resistance exist in the literature, all definitions have in common that resistance is based on genetic changes introduced by mutations or horizontal gene transfer and thus are heritable traits [42]. In reference to the study by Krewing *et al*. [43], wherein *E. coli* strains that differ genetically were analysed (i.e. deletion mutants or over-producing strains), we here analysed *hslO* mutants with regard to the level of resistance conferred by the genetic differences to the isogenic parental strain. One of the known effects of plasma treatment is protein aggregation, *in vivo* as well as *in vitro* [7,14]. In a previous study, plasma sensitivities of the deletion strain Δ*hslO* and the complemented strain were determined [14]. Since in that study a plasma source designed for nail parlours was used, that does not lend itself to mechanistic studies, the sensitivity of *E. coli* Δ*hslO* and the complemented strain was tested here using the clinically relevant PlasmaDerm DBD which is widely used, i.e. in dermatology, and has been well-characterized (reviewed in [44]). The *E. coli* wild-type BW25113 [45], the deletion mutant Δ*hslO* [45] (both with or without the empty vector plasmid pCA24N), and the complemented strain Δ*hslO* + pCA24N::*hslO* [28] expressing His_6_-tagged Hsp33 IPTG-dependently were exposed to the PlasmaDerm-generated plasma for 60 s. To correlate changes in plasma resistances to protein levels of the complemented strain, western blot analyses were performed (figure 1*a*). *E. coli* wild-type exhibited a survival rate of approximately 65%, which decreased to 50% in the strain containing the empty vector. The deletion strain Δ*hslO* showed a decreased survival rate of 35%, but in contrast to the wild-type, in the Δ*hslO* strain no effect of the empty vector was detectable. Complementation without addition of IPTG led to survival rates comparable to those of the wild-type, indicating that the amount of Hsp33 produced from the leaky promoter of the pCA24N-plasmid might be comparable to the amount of Hsp33 in the wild-type. The survival rates after plasma treatment increased with increasing IPTG concentrations, which correlated with increasing signal intensities in the western blot. The highest survival rate (95%) was reached when inducing with 1000 µM IPTG.

Since an over-production of *hslO* led to higher survival rates upon plasma treatment compared to the wild-type, prolonged plasma treatment times were tested to assess if the complemented strain induced with 1000 µM IPTG survives plasma treatment for longer than the wild-type (figure 1*b*). Differences in survival were detectable between the wild-type and the Hsp33 over-production strain in the time span from 60 to 120 s with survival rates of the over-producing strain exceeding those of the wild-type up to three-fold. However, after 180 s plasma treatment no survival was observed for either strain.

### 3.2. Hsp33 is degraded by plasma

**Figure 2.**
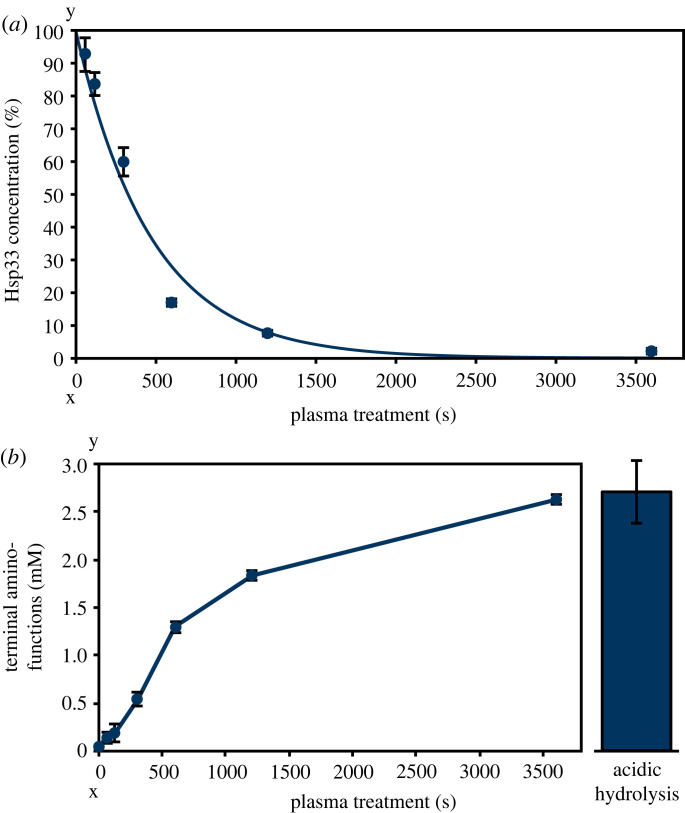
Plasma stability of Hsp33. (*a*) Protein concentration of Hsp33 as a function of different plasma treatment times. 1 mg ml^−1^ Hsp33 was treated with plasma for indicated times and Bradford reagent was used to determine the protein concentration of the treated solutions. Concentration of an untreated sample was set to 100%. (*b*) Terminal amino functions as a function of different plasma treatment times were determined using the ninhydrin assay. After treatment of 1 mg ml^−1^ Hsp33 with plasma ninhydrin was added, which reacts with terminal amino functions resulting in an increased absorption at 575 nm. As a control, acidic hydrolysis of Hsp33 was performed to obtain signal intensities for maximal degradation. (*a,b*) Means and standard deviations represent three experiments.

*In vivo* Hsp33 provided a notable benefit regarding bacterial plasma survival; thus, the impact of plasma on purified protein *in vitro* was characterized focusing on the activation mechanism. In previous studies it was shown that plasma treatment leads to degradation of proteins [5,12]. Thus, the effect of plasma treatment on Hsp33 stability was analysed by determining the protein concentration using Bradford reagent after different plasma treatment times (figure 2*a*). Here, we detected a fast decrease of the Hsp33 concentration within the first 600 s of plasma treatment (17%). Longer treatment times (up to 3600 s) led to complete degradation of Hsp33. Plasma-induced protein degradation goes along with formation of new terminal amino functions, which are detectable using ninhydrin [12]. An increase in terminal amino functions after different plasma treatment times was observed (figure 2*b*). As a positive control and for quantifying maximal degradation of Hsp33, acidic hydrolysis of Hsp33 was conducted to completely degrade the protein to its amino acids. Degradation of Hsp33 by acidic hydrolysis led to a comparable increase in terminal amino functions to those observed after 3600 s plasma treatment, showing that Hsp33 is degraded by plasma.

### 3.3. Plasma-induced activation of Hsp33

**Figure 3.**
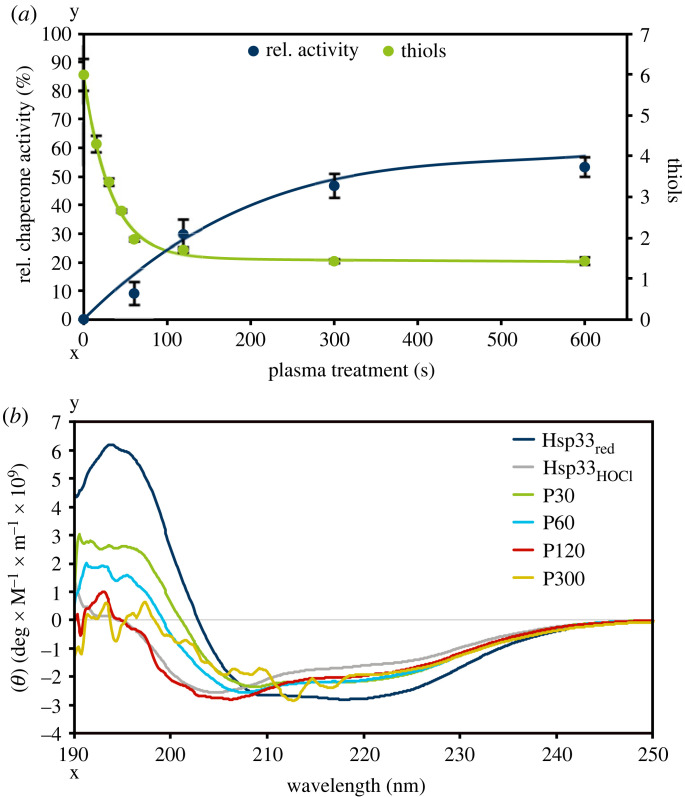
Plasma-induced Hsp33 activation. (*a*) Determination of relative chaperone activity and thiol oxidation after different plasma treatment times. The relative chaperone activity was determined by a citrate synthase-based assay, to which aggregate-free Hsp33 was added. Activity of Hsp33_red_ was set to 0%, activity of Hsp33_HOCl_ was set to 100%. The thiol oxidation state was determined by incubation of plasma-treated Hsp33 with Ellman's reagent. Afterwards, absorption at 412 nm was measured. Means and standard deviations representing three experiments are shown. (*b*) Far-UV CD spectra of Hsp33_red_, Hsp33_HOCl_, and plasma treated Hsp33. Hsp33 was treated with plasma for 30 (P30), 60 (P60), 120 (P120), and 300 s (P300) and measured immediately. The data reflect an average of three independent experiments.

The previous study of Krewing *et al.* [14] showed that Hsp33 is activated by plasma using a SoftBond DBD which causes oxidative disulfide bond formation and protein unfolding. To validate this effect for the DBD used in this study, plasma-induced activation was tested using the citrate synthase assay (figure 3*a*). Adding plasma-treated Hsp33 to the citrate synthase assay already increased the observed light scattering, indicating that Hsp33 itself aggregates upon plasma treatment. Thus, for all following experiments, the protein solution was centrifuged after plasma treatment at 15 000 rpm for 45 min to remove Hsp33 aggregates. Of the aggregate-free supernatant, 900 nM were applied in the chaperone activity assay, as determined by Bradford assay (figure 3*a*).

For plasma treatment times up to 300 s, a steady increase of chaperone activity was observed reaching an activity of approximately 50% compared to HOCl-oxidized Hsp33. Longer treatment for up to 600 s did not lead to a further increase in activity. However, it was confirmed that plasma treatment using the PlasmaDerm DBD activates Hsp33.

Besides activation, plasma-induced thiol oxidation of Hsp33 was validated using the Ellman's assay [31] (figure 3*a*). Without plasma treatment, as expected, an average of six reduced thiols were detectable. After 60 s of plasma treatment, on average only one reduced thiol was detected. One thiol function remained, even after 600 s of treatment. Activation of Hsp33 relies on conformational changes. Thus, activation by the PlasmaDerm DBD was investigated using CD spectroscopy (figure 3*b*) to assess protein unfolding. Plasma treatment of 30 and 60 s led to intermediate unfolding compared to Hsp33_red_ and Hsp33_HOCl_, while Hsp33, which was treated for 120 s, showed an unfolding that was comparable to that of Hsp33_HOCl_. However, prolonged plasma treatment (300 s) resulted in an even stronger unfolding of Hsp33, while the noise in the spectra increased, indicating that the sample contained a mixture of undefined structures.

### 3.4. Plasma induces different Hsp33 modifications

**Figure 4.**
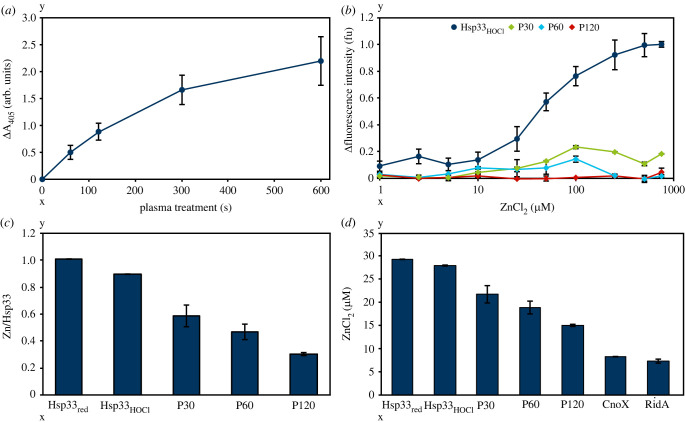
Plasma-induced modifications of Hsp33. (*a*) Carbonylation level of plasma-treated Hsp33 as measured mixing DNPH with Hsp33. The absorption at 405 nm was measured and corrected for untreated Hsp33. (*b*) Zinc-induced conformational changes of Hsp33_HOCl_ or plasma-oxidized Hsp33 (P30, P60, P120) by following the changes in tryptophan fluorescence at 340 nm. (*c*) Zinc binding ability of chemically (Hsp33_HOCl_) or plasma-oxidized (P30, P60, P120) Hsp33 using the zincon assay. (*d*) Zinc-binding ability of Hsp33 using the zincon assay. Reduced (Hsp33_red_), chemically oxidized (Hsp33_HOCl_) and plasma-treated (P30, P60, P120) Hsp33 were investigated. Proteins CnoX and RidA served as controls for unspecific ZnCl_2_ binding. (*a–d*) Means and standard deviations representing three experiments are shown.

Oxidation of several amino acid residues in proteins due to plasma treatment are well-known modifications [46,47]. To verify that in addition to cysteine oxidation, Hsp33 suffers from further oxidation by plasma, the degree of carbonylation was investigated using DNPH as an indicator of oxidative damage [48]. To this end, DNPH was added to plasma-treated Hsp33. An increase in the absorption at 405 nm was observed, which points to oxidative modifications of amino acid side chains upon plasma treatment (figure 4*a*).

The chemical activation of Hsp33 by HOCl *in vitro* is reversible by incubating the oxidized protein with DTT and zinc ions [22]. Moreover, Krewing *et al*. [14] showed that plasma-induced activation of Hsp33 is reversible as well. However, no complete refolding of the plasma-treated and subsequently reduced Hsp33 was detected. This effect was also observed when using the PlasmaDerm DBD source (electronic supplementary material, figure S3). The unspecific oxidation of Hsp33 after plasma treatment (figure 4*a*) and the lack of complete refolding after overnight reduction raised the question as to whether or not the partially refolded Hsp33 is still able to bind zinc with its four conserved thiols. To this end, the zinc-binding ability of plasma-treated Hsp33 was analysed by following the conformational changes during incubation with zinc ions based on tryptophan fluorescence. Incubation of HOCl-oxidized Hsp33 with 25 µM ZnCl_2_ led to a conformational change, while plasma-treated protein did not show any structural change at this zinc concentration. Plasma-treated Hsp33 (treated for 30 or 60 s) showed a slight increase in fluorescence intensity after an incubation with 100 µM ZnCl_2_; however, it was only 20% compared to 100% in chemically activated Hsp33. Since this effect might be due to plasma-induced modifications of tryptophan, the zinc-binding ability was investigated in a second approach using zincon [34]. The non-zinc-binding proteins CnoX_Ec_ and RidA_Ec_ were also tested for their zinc-binding property, enabling us to estimate unspecific zinc binding by proteins, e.g. through amino acid side chains like histidine (figure 4*d*). For chemically oxidized Hsp33 (Hsp33_HOCl_) on average one zinc ion was coordinated by one Hsp33 molecule after overnight reduction, while plasma treatment of 30 s already resulted in a decrease in zinc binding ability: on average 0.6 zinc ions were bound per Hsp33 molecule (figure 4*c*). Moreover, prolonged treatment times decreased the amount of bound zinc per Hsp33 molecule even further. Using this approach, the finding of a negatively influenced zinc-binding ability of Hsp33 as determined by means of tryptophan fluorescence (figure 4*b*) was confirmed.

### 3.5. Hsp33 is activated by the plasma-produced species superoxide, singlet oxygen and atomic oxygen

To gain insights as to which plasma-generated oxygen-dependent species are responsible for activating Hsp33, different species were generated *in vitro* for incubation with Hsp33 at room temperature or 43°C, since Hsp33 is known to be activated by fast-acting oxidants at room temperature and by slow-acting oxidants in combination with elevated temperature [20–22].

**Figure 5.**
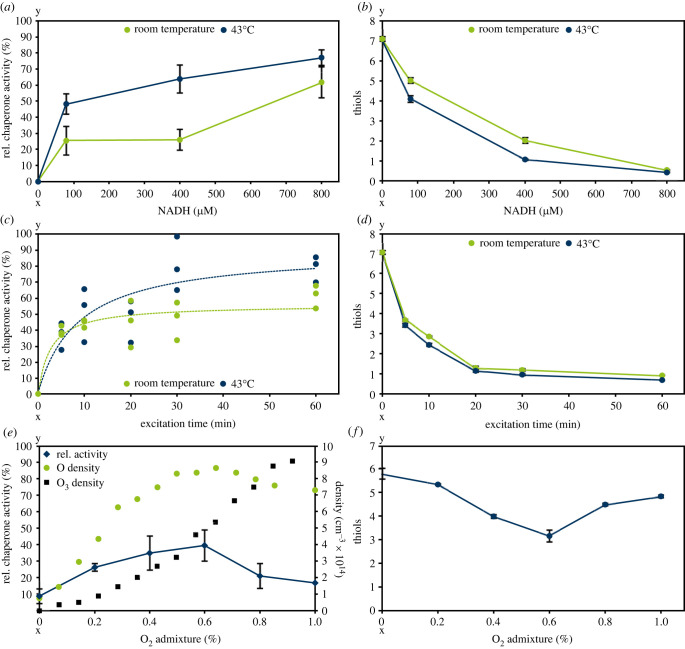
Hsp33 activation by superoxide (*a,b*), singlet oxygen (*c,d*), and atomic oxygen (*e,f*). (*a,b*) Relative chaperone activity and thiol oxidation upon addition of different amounts of NADH (80, 400 and 800 µM). Incubation of Hsp33 with the reaction mixture (NBT, NADH and PMS) was carried out at room temperature (green) or 43°C (blue). (*c,d*) Relative chaperone activity and thiol oxidation at different excitation times of methylene blue, leading to singlet oxygen formation. The incubation of Hsp33 with singlet oxygen was carried out at room temperature (green) or 43°C (blue). (*e,f*) Hsp33 was treated for 30 s with the effluent of the plasma jet, while the oxygen content was varied (0–1% O_2_). Variations in oxygen admixture lead to production of different amounts of atomic oxygen [26]. Afterwards, chaperone activity and thiol oxidation were determined. Data on atomic oxygen and ozone generation were taken from [26]. (*a,b,d*–*f*) Means and standard deviations reflect three experiments; (*c*) variation was high and the data for three replicates are shown.

Superoxide ${(O}_{2}^{-})$ was generated by using NADH, PMS, and NBT at room temperature or 43°C. In this reaction, NADH is consumed, and superoxide is formed stoichiometrically from molecular oxygen using PMS as catalyst. The consumption of NADH was followed at 340 nm. Additionally, the $O_{2}^{-}$ production was observed by adding NBT to the assay, which reacts with $O_{2}^{-}$ under formation of a blue product absorbing at 560 nm (electronic supplementary material, figure S1). Thus, in control experiments without Hsp33, decreased absorption at 340 nm and an increased absorption at 560 nm showed superoxide formation. At room temperature, Hsp33 showed chaperone activity of about 25% when 80 µM or 400 µM NADH was added to the reaction mixture. Application of 800 µM NADH led to an Hsp33 activity of approximately 60%. By contrast, the observed activities of Hsp33 incubated with the superoxide-generating reaction mixture at 43°C were higher than those incubated at room temperature (figure 5*a*). Furthermore, thiol oxidation by superoxide was determined, showing a complete oxidation of Hsp33 at an addition of 800 µM NADH (figure 5*b*).

Singlet oxygen was produced by exciting methylene blue at 606 nm for different durations together with Hsp33 [38,39]. Incubation with singlet oxygen led to an activation of Hsp33 at both room temperature and 43°C, with a higher activation rate at 43°C. Moreover, thiol oxidation of Hsp33 correlated with its activation kinetics at room temperature and 43°C (figure 5*c*,*d*).

**Figure 6.**
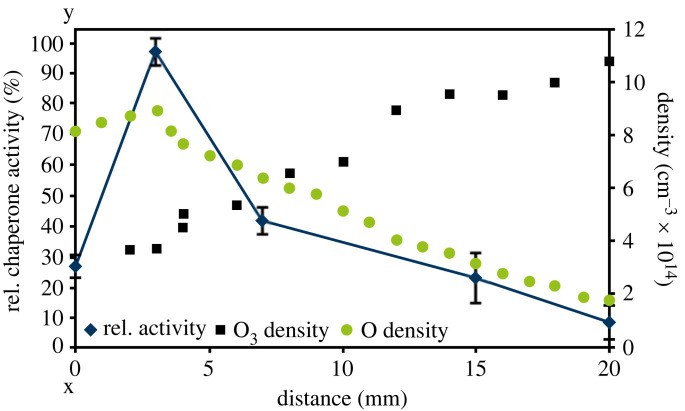
Distance-dependent activation of Hsp33 activation using the µAPPJ plasma jet. Hsp33 was treated for 30 s with the effluent of the plasma jet, while the distance was varied (0–20 mm). This variation of the distance leads to treatment with different densities of atomic oxygen and ozone [26]. Afterwards, chaperone activity was measured. Data on atomic oxygen and ozone generation were taken from [26]. Means and standard deviations representing three experiments are shown.

Ellerweg *et al*. showed for a microscale atmospheric plasma jet (µAPPJ) a dependency of atomic oxygen and ozone production on the oxygen admixture to the feed gas [26]. To test if Hsp33 activation by plasma might correlate with formation of atomic oxygen, the plasma jet operated with different oxygen admixtures was used for Hsp33 treatment (figure 5*e*,*f*). Activity of Hsp33 was higher when treated with increasing O_2_ admixtures, leading to a maximal activity at an admixture of 0.6% O_2_. A further increase of O_2_ led to decreased activity. The increase in activity correlated with the atomic oxygen production, which also peaked at 0.6% (figure 5*e*) [26]. Investigation of thiol oxidation at different oxygen admixtures to the feed gas showed a correlation to the activation kinetics of Hsp33. Admixtures of 0.8% and 1% oxygen, however, led to an oxidation state comparable to that for an admixture of 0.2% oxygen, while activity was lower at these admixtures. In a different experiment, the distance of the nozzle of the jet to the sample was varied, also leading to different densities of atomic oxygen and ozone (figure 6) [26]. Hsp33 activation reached its maximum at a distance of 3 mm, correlating with the highest atomic oxygen density [26]. Increasing the distance leads to lower atomic oxygen and higher ozone densities [26], rendering Hsp33 inactive. While the chaperone activity correlated with the density of atomic oxygen, it cannot be excluded that in the plasma jet experiments this effect may be caused by other species, particularly superoxide and/or singlet oxygen, the density profiles of which have not been reported to the best of our knowledge.

**Figure 7.**
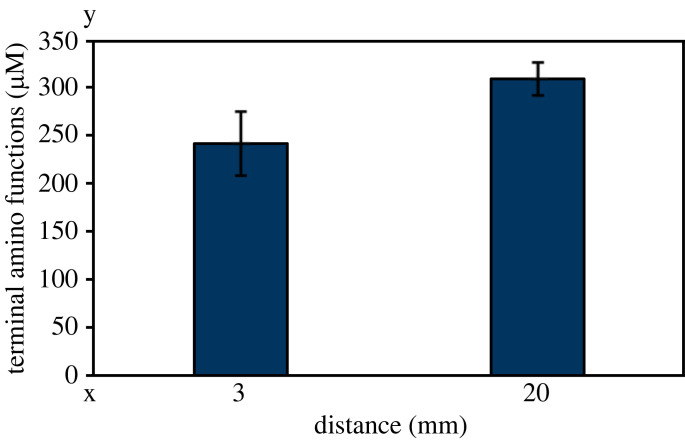
Ozone-dependent Hsp33 degradation. Hsp33 was treated for 60 s with the effluent of the plasma jet at either 3 or 20 mm distance from the end of the nozzle of the jet. Afterwards, ninhydrin was added to quantify terminal amino functions. Means and standard deviations of three experiments are shown.

Taking the higher ozone densities at higher oxygen admixtures into account, the question arises as to whether the increased ozone densities have a negative influence on Hsp33 activation. To address this, degradation of Hsp33 was investigated after treatment with the plasma jet using short (low ozone density) and high distances (high ozone density), revealing a slightly increased degradation of Hsp33 at high ozone densities (figure 7).

**Figure 8.**
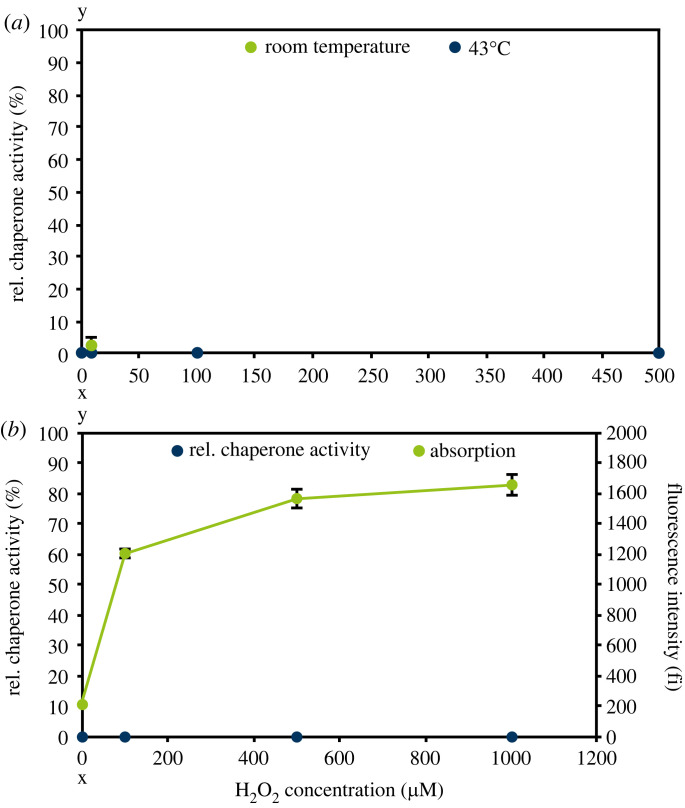
Investigation of Hsp33 activation upon treatment with peroxynitrite (*a*) and hydroxyl radicals (*b*). (*a*) Relative chaperone activities at different amounts of peroxynitrite added are displayed. Incubation of Hsp33 with peroxynitrite was carried out at room temperature or 43°C. (*b*) Relative chaperone activity (blue) and fluorescence intensities of produced HTA at 425 nm (green) at different hydrogen peroxide concentrations are displayed. The Fenton reaction was used to generate hydroxyl radicals. 0.5 mM FeSO_4_ was incubated together with up to 10 mM H_2_O_2_ and Hsp33 for 1 h. Formation of hydroxyl radicals was monitored using terephthalic acid, which reacts with hydroxyl radicals to HTA leading to a fluorescence signal (*λ*_ex_ = 315 nm; *λ*_em_ = 425 nm). The incubation of Hsp33 with hydroxyl radicals was carried out at room temperature. (*a,b*) Means and standard deviations of three experiments are shown.

Peroxynitrite and hydroxyl radicals were also tested for their ability to activate Hsp33 (figure 8). Peroxynitrite was chemically synthesized and added in defined amounts to Hsp33 [37]. Hydroxyl radicals were generated from FeSO_4_ and H_2_O_2_ using the Fenton reaction [40], while its formation was detected using terephthalic acid [41]. An incubation of Hsp33 with these two reactive species did not lead to an activation, excluding them as Hsp33 activators.

**Figure 9.**
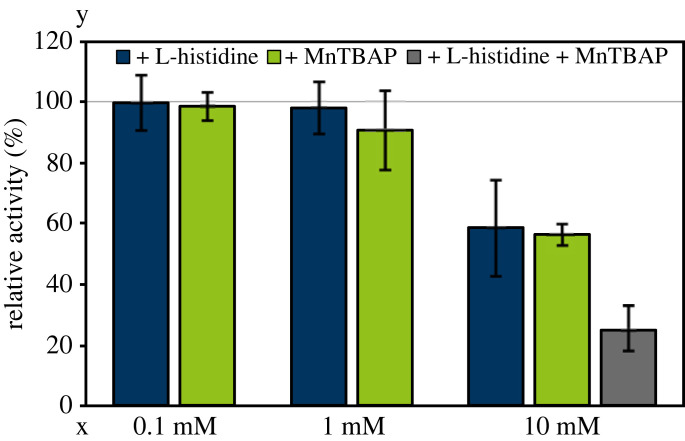
Plasma-induced Hsp33 activation in the presence of scavengers. Hsp33 was treated for 120 s in presence of different concentrations of L-histidine (blue) and MnTBAP (green). Activity without scavenger addition was set to 100%. Combination of both scavengers was also conducted (grey) with 10 mM of each scavenger. Means and standard deviations reflecting three experiments are shown.

To further validate superoxide and singlet oxygen as potential plasma-generated activators of Hsp33, scavengers of these species were used. To this end, plasma treatment of Hsp33 was conducted together with MnTBAP to scavenge superoxide [49] and/or L-histidine to scavenge singlet oxygen [50]. Concentrations of up to 1 mM did not show any influence on plasma-induced Hsp33 activation when treated for 120 s with plasma (figure 9). Nonetheless, addition of 10 mM of the individual scavenger decreased Hsp33 activity by 50%. Combination of both scavengers (each with 10 mM) led to a further decrease; only 25% activity was observed compared to Hsp33 treatment without scavengers.

## 4. Discussion

### 4.1. Hsp33 mediates plasma resistance

Plasma is already used in a variety of medical and biological applications, but the fundamentals underlying bacterial inactivation are not yet fully understood. Moreover, to date it is still unclear as to whether bacterial resistance against plasma might develop. In previous studies, heat shock and aggregation-preventing proteins were shown to be crucial for bacterial plasma survival [12,43]. Using a quantitative CFU-based assay, we further elucidated the influence of Hsp33 on bacterial survival of plasma treatment (figure 1*a*). The deletion mutant Δ*hslO* lacking Hsp33 displayed an increased plasma sensitivity compared to the wild-type, while over-production of Hsp33 caused plasma survival rates to increase above wild-type levels. Thus, Hsp33 seems to contribute to bacterial plasma resistance. Prolonged plasma treatment times were applied, revealing higher survival rates of the Hsp33 over-production strain even at longer treatment times indicating that initially protein aggregation significantly contributes to bacterial inactivation (figure 1*b*). However, a treatment of 180 s resulted in complete inactivation of both the wild-type and the Hsp33 over-production strain. At that time, likely other mechanisms become overwhelming.

### 4.2. Plasma-induced Hsp33 degradation and aggregation

Hsp33 is an exceptional protein regarding its interaction with plasma, since unlike most other proteins it is activated by plasma [5,9,14,51]. Here, we confirmed not only plasma-induced activation of Hsp33 at short treatment times (figure 3*a*) but also detected degradation and aggregation at longer treatment times. A decrease in protein concentration within the first 600 s of plasma treatment was observed (figure 2*a*). Plasma-induced protein degradation appears to be due to cleavage of peptide bonds, resulting in the formation of new terminal amino functions [12]. We identified an increase in terminal amino functions with increasing plasma treatment times of Hsp33, confirming plasma-induced Hsp33 degradation (figure 2*b*).

In addition to degradation, when adding plasma-treated Hsp33 to the citrate synthase assay, we observed an increase in light scattering, pointing to aggregation of Hsp33 during plasma exposure.

In general, three competitive processes seem to occur upon plasma treatment of Hsp33. The first and fastest causes activation of the molecular chaperone Hsp33. With increasing treatment times, more Hsp33 molecules are activated by plasma, reaching a maximum after 300 s treatment. The second is the degradation of the protein into peptides. And, finally, the third process is an aggregation that likely occurs due to modifications of amino acid residues relevant for the protein structure. Modifications of amino acids indeed result from plasma treatment as shown for the increased carbonylation of Hsp33 upon plasma treatment (figure 4*a*). Aggregation of Hsp33 slows degradation, likely because aggregates are less accessible to species causing degradation [14].

### 4.3. Plasma influences the zinc-binding ability of Hsp33

Reversibility of plasma-induced activation of Hsp33 was observed; however, complete refolding of the protein into its native state known to occur under physiological conditions was not detectable [14]. Within this study, we verified the inability of refolding of plasma-treated Hsp33 after reduction (electronic supplementary material, figure S2), leading to the question as to whether or not the plasma-activated and subsequently reduced Hsp33 still binds a zinc ion with its four conserved cysteines. To this end, the ability of plasma-treated Hsp33 to bind zinc was determined using two approaches. First, we investigated conformational changes on the basis of tryptophan fluorescence, revealing a conformational change of Hsp33_HOCl_ after incubation with 25 µM ZnCl_2_, while no comparable conformational change was detected for plasma-treated Hsp33 (figure 4*b*). However, we cannot exclude that this effect is due to plasma-induced modifications of tryptophan. Thus, a second approach was chosen using zincon to detect Hsp33-bound zinc (figure 4*c*). Indeed, plasma treatment of Hsp33 decreased the average amount of bound zinc ions per Hsp33 molecule compared to untreated Hsp33. The decreased zinc-binding ability of plasma-exposed Hsp33 was not based on modifications of the zinc coordinating cysteines, since it was shown that they were completely reduced after overnight incubation (electronic supplementary material, figure S2) [14]. Presumably, the fact that Hsp33 does not refold into its native state after plasma treatment adversely affects the zinc-binding ability. However, despite not binding zinc, plasma-treated and subsequently reduced Hsp33 was shown to be inactive, supporting the hypothesis that zinc binding only plays a minor role in regulating Hsp33 activity. This is consistent with previous studies showing that zinc release alone is not sufficient for Hsp33 activation [23] and that an oxidized, zinc-free Hsp33 monomer was also partially active [32].

### 4.4. Offset of plasma-induced Hsp33 activation

We showed that plasma-induced activation of Hsp33 relies on both thiol oxidation and protein unfolding. Hsp33 was completely oxidized after 60 s plasma treatment, while activity increased significantly after 120 s (figure 3*a*). This is consistent with the results from CD spectroscopy, since unfolding of Hsp33 reached the Hsp33_HOCl_ level not until 120 s of plasma treatment, indicating that there is an offset between oxidation of the redox switch domain and activation of Hsp33 due to unfolding of its linker region. As previously reported, unfolding of the protein is a prerequisite for Hsp33 dimerization and activation, since the linker region masks the substrate binding site when Hsp33 is not unfolded [20,32]. Our results confirmed that plasma-induced activation of Hsp33 is highly dependent on unfolding of its linker region.

Plasma-treated and subsequently reduced Hsp33 did not refold into its native conformation although its thiols were reduced and the protein was inactive. Moreover, a second plasma treatment resulted in an oxidation of the thiols and an active protein, for which even further unfolding was detectable (electronic supplementary material, figure S2) [14]. The reversibility of activation by thiol reduction points to a potentially new model of protein activity regulation. Previously a dependency of Hsp33 activation on protein unfolding was described [17,20,25]. However, when treated with plasma, likely major parts of the protein unfold and cannot refold properly, while the linker region still refolds into its native state upon reduction and masks the substrate binding site of Hsp33. Thus, a subsequent plasma-induced activation relies again on the unfolding of the linker region, leaving the substrate binding site uncovered for protein binding.

### 4.5. Plasma-induced activation of Hsp33 may depend on superoxide, singlet oxygen, and atomic oxygen

The molecular chaperone Hsp33 was shown to be activated by direct plasma treatment *in vitro*. However, little is known about plasma-generated species responsible for Hsp33 activation. Previous studies on Hsp33 showed that the chaperone is activated by HOCl treatment or by treatment with H_2_O_2_ or •NO when combined with unfolding conditions, e.g. elevation of temperature to 43°C [18,22]. Measurements of HOCl in plasma-treated liquids revealed that concentrations were too low to explain Hsp33 activation. In this earlier study, no activation of Hsp33 was observed in an oxygen-free atmosphere. Therefore, an oxygen-dependent activator was postulated [14]. In this study, the PlasmaDerm DBD of Cinogy was used for plasma treatment. Since this source is used for treating patients, it is designed to avoid heating of treated samples, and it has been shown that during a 600 s treatment the substrate stays at room temperature [52]. Although DBD treatment does produce H_2_O_2_ and •NO, it is questionable if physical properties of plasma create protein unfolding conditions that together with H_2_O_2_ or •NO result in Hsp33 activation. It appeared more likely that other plasma-generated reactive oxygen species are capable of activating Hsp33. To test this, six known reactive oxygen species (superoxide, singlet oxygen, atomic oxygen, ozone, peroxynitrite and hydroxyl radicals) were investigated for their effect on Hsp33 activity.

While peroxynitrite at room temperature or 43°C and hydroxyl radicals at room temperature did not cause Hsp33 activation (figure 8), superoxide effectively activated Hsp33 at room temperature (figure 5*a*) and thiol oxidation was also observed (figure 5*b*). To our knowledge it has not been described previously that thiols of zinc finger motifs, comparable to those of the redox switch domain of Hsp33, react with superoxide. Moreover, superoxide is thought to play a minor role in bacterial inactivation by plasma compared to other ROS due to its relatively low redox potential and inability to cross the cell membrane [53,54]. Superoxide reacts with hydrogen peroxide-forming hydroxyl radicals (Haber–Weiss reaction) [55]. This cascade has been shown to effectively inactivate bacteria [43]. Since hydroxyl radicals did not activate Hsp33 *in vitro* (figure 8), this cascade likely does not play a significant role in Hsp33 activation. Superoxide is one of the first species generated upon oxidative stress, e.g. in macrophages [56], and it would be of benefit to the bacterium if Hsp33 were rapidly activated by this early species for protecting the cell from protein aggregation. By activating Hsp33, superoxide could act as a signal to the bacterium, warning of further oxidative stress.

Besides superoxide, activation of Hsp33 by treatment with singlet oxygen was observed *in vitro* (figure 5*c*). This is consistent with the previously postulated interaction of singlet oxygen with zinc finger motifs comparable to the redox switch domain of Hsp33 [57]. Singlet oxygen is generated by plasma, making it a plausible plasma-generated Hsp33 activator. Scavengers of singlet oxygen and superoxide led to decreased Hsp33 activation, when adding them at a 300-fold molar excess during plasma treatment (figure 9). A combination of both scavengers further decreased Hsp33 activity. Depending on the selectivity of the scavengers for singlet oxygen and superoxide over other reactive species, these results may confirm an involvement of singlet oxygen and superoxide in plasma activation of Hsp33.

Using the microscale atmospheric pressure plasma jet (µAPPJ) [26], we investigated the dependency of Hsp33 activation on atomic oxygen. Here, a correlation between Hsp33 activation and atomic oxygen density was observed (figure 5*e*). Since the distance between the nozzle of the jet and the sample was less than 5 mm, this effect did not rely on ozone which forms only at greater distance (figure 6) [26]. Thiol oxidation of Hsp33 also correlated with atomic oxygen density (figure 5*f*). However, thiol oxidation was still observed at high oxygen admixtures (0.8–1%) while activation decreased significantly. This effect was also observed when increasing the distance of the nozzle of the jet to the sample, resulting in increasing ozone densities (figure 6). Thus, the previously mentioned decrease in Hsp33 activity might be due to the increased ozone densities [26] inactivating Hsp33 (figure 7). The chaperone activity correlated with the density of atomic oxygen, but not with ozone densities. Thus, while ozone can be ruled out as potential activator, atomic oxygen remains a good candidate activator in addition to superoxide and singlet oxygen.

We thus conclude based on testing individual reactive species, scavenger experiments and correlations of Hsp33 activation and species density profiles that singlet oxygen and superoxide, and potentially atomic oxygen, contribute to Hsp33 activation by plasma treatment while ozone has rather detrimental effects.

## Data Availability

The data are provided in electronic supplementary material [58].

## References

[1] Ehlbeck J, Schnabel U, Polak M, Winter J, von Woedtke T, Brandenburg R, von dem Hagen T, Weltmann K-D. 2011 Low temperature atmospheric pressure plasma sources for microbial decontamination. J. Phys. D: Appl. Phys. **44**, 013002. (10.1088/0022-3727/44/1/013002)

[2] Halfmann H, Denis B, Bibinov N, Wunderlich J, Awakowicz P. 2007 Identification of the most efficient VUV/UV radiation for plasma based inactivation of *Bacillus atrophaeus* spores. J. Phys. D: Appl. Phys. **40**, 5907. (10.1088/0022-3727/40/19/019)

[3] Privat-Maldonado A, O'Connell D, Welch E, Vann R, van der Woude MW. 2016 Spatial dependence of DNA damage in bacteria due to low-temperature plasma application as assessed at the single cell level. Sci. Rep. **6**, 35646. (10.1038/srep35646) 27759098 PMC5069486

[4] Choi S, Attri P, Lee I, Oh J, Yun J-H, Park JH, Choi EH, Lee W. 2017 Structural and functional analysis of lysozyme after treatment with dielectric barrier discharge plasma and atmospheric pressure plasma jet. Sci. Rep. **7**, 1027. (10.1038/s41598-017-01030-w) 28432354 PMC5430822

[5] Deng XT, Shi JJ, Kong MG. 2007 Protein destruction by a helium atmospheric pressure glow discharge: capability and mechanisms. J. Appl. Phys. **101**, 074701. (10.1063/1.2717576)

[6] Lackmann J-W, Schneider S, Edengeiser E, Jarzina F, Brinckmann S, Steinborn E, Havenith M, Benedikt J, Bandow JE. 2013 Photons and particles emitted from cold atmospheric-pressure plasma inactivate bacteria and biomolecules independently and synergistically. J. R Soc. Interface **10**, 20130591. (10.1098/rsif.2013.0591) 24068175 PMC3808546

[7] Itooka K, Takahashi K, Kimata Y, Izawa S. 2018 Cold atmospheric pressure plasma causes protein denaturation and endoplasmic reticulum stress in *Saccharomyces cerevisiae*. Appl. Microbiol. Biotechnol. **102**, 2279-2288. (10.1007/s00253-018-8758-2) 29356871

[8] Takai E, Kitano K, Kuwabara J, Shiraki K. 2012 Protein inactivation by low-temperature atmospheric pressure plasma in aqueous solution. Plasma Process Polym. **9**, 77-82. (10.1002/ppap.201100063)

[9] Lackmann J-W et al. 2015 A dielectric barrier discharge terminally inactivates RNase A by oxidizing sulfur-containing amino acids and breaking structural disulfide bonds. J. Phys. D: Appl. Phys. **48**, 494003. (10.1088/0022-3727/48/49/494003)

[10] Segat A, Misra NN, Cullen PJ, Innocente N. 2015 Atmospheric pressure cold plasma (ACP) treatment of whey protein isolate model solution. Innov. Food Sci. Emerg. **29**, 247-254. (10.1016/j.ifset.2015.03.014)

[11] Lee HJ, Shon CH, Kim YS, Kim S, Kim GC, Kong MG. 2009 Degradation of adhesion molecules of G361 melanoma cells by a non-thermal atmospheric pressure microplasma. New J. Phys. **11**, 115026. (10.1088/1367-2630/11/11/115026)

[12] Krewing M, Schubert B, Bandow JE. 2020 A dielectric barrier discharge plasma degrades proteins to peptides by cleaving the peptide bond. Plasma Chem. Plasma Process. **40**, 685-696. (10.1007/s11090-019-10053-2)

[13] Chuang SE, Blattner FR. 1993 Characterization of twenty-six new heat shock genes of *Escherichia coli*. J. Bacteriol. **175**, 5242-5252. (10.1128/jb.175.16.5242-5252.1993) 8349564 PMC204992

[14] Krewing M et al. 2019 The molecular chaperone Hsp33 is activated by atmospheric-pressure plasma protecting proteins from aggregation. J. R. Soc. Interface **16**, 20180966. (10.1098/rsif.2018.0966) 31213177 PMC6597770

[15] Jakob U, Muse W, Eser M, Bardwell JC. 1999 Chaperone activity with a redox switch. Cell **96**, 341-352. (10.1016/s0092-8674(00)80547-4) 10025400

[16] Kim S-J, Jeong D-G, Chi S-W, Lee J-S, Ryu S-E. 2001 Crystal structure of proteolytic fragments of the redox-sensitive Hsp33 with constitutive chaperone activity. Nat. Struct. Biol. **8**, 459-466. (10.1038/87639) 11323724

[17] Groitl B, Horowitz S, Makepeace KAT, Petrotchenko EV, Borchers CH, Reichmann D, Bardwell JCA, Jakob U. 2016 Protein unfolding as a switch from self-recognition to high-affinity client binding. Nat. Commun. **7**, 10357. (10.1038/ncomms10357) 26787517 PMC4735815

[18] Jakob U, Eser M, Bardwell JCA. 2000 Redox switch of Hsp33 has a novel zinc-binding motif. J. Biol. Chem. **275**, 38 302-38 310. (10.1074/jbc.m005957200) 10976105

[19] Janda I et al. 2004 The crystal structure of the reduced, Zn^2+^-bound form of the *B. subtilis* Hsp33 chaperone and its implications for the activation mechanism. Structure **12**, 1901-1907. (10.1016/j.str.2004.08.003) 15458638 PMC3691021

[20] Cremers CM, Reichmann D, Hausmann J, Ilbert M, Jakob U. 2010 Unfolding of metastable linker region is at the core of Hsp33 activation as a redox-regulated chaperone. J. Biol. Chem. **285**, 11 243-11 251. (10.1074/jbc.m109.084350) PMC2857002 20139072

[21] Ilbert M, Horst J, Ahrens S, Winter J, Graf PCF, Lilie H, Jakob U. 2007 The redox-switch domain of Hsp33 functions as dual stress sensor. Nat. Struct. Mol. Biol. **14**, 556-563. (10.1038/nsmb1244) 17515905 PMC2782886

[22] Winter J, Ilbert M, Graf PCF, Özcelik D, Jakob U. 2008 Bleach activates a redox-regulated chaperone by oxidative protein unfolding. Cell **135**, 691-701. (10.1016/j.cell.2008.09.024) 19013278 PMC2606091

[23] Graumann J, Lilie H, Tang X, Tucker KA, Hoffmann JH, Vijayalakshmi J, Saper M, Bardwell JCA, Jakob U. 2001 Activation of the redox-regulated molecular chaperone Hsp33: a two-step mechanism. Structure **9**, 377-387. (10.1016/s0969-2126(01)00599-8) 11377198

[24] Chi S-W, Jeong DG, Woo JR, Lee HS, Park BC, Kim BY, Erikson RL, Ryu SE, Kim SJ. 2011 Crystal structure of constitutively monomeric *E. coli* Hsp33 mutant with chaperone activity. FEBS Lett. **585**, 664-670. (10.1016/j.febslet.2011.01.029) 21266175

[25] Hoffmann JH, Linke K, Graf PCF, Lilie H, Jakob U. 2004 Identification of a redox-regulated chaperone network. EMBO J. **23**, 160-168. (10.1038/sj.emboj.7600016) 14685279 PMC1271656

[26] Ellerweg D, Benedikt J, von Keudell A, Knake N, der Gathen VS. 2010 Characterization of the effluent of a He/O_2_ microscale atmospheric pressure plasma jet by quantitative molecular beam mass spectrometry. New J. Phys. **12**, 013021. (10.1088/1367-2630/12/1/013021)

[27] Sambrook J, Russel DW. 2001 *Molecular cloning: a laboratory manual*, 3rd edn. New York, NY: CSHL Press.

[28] Kitagawa M, Ara T, Arifuzzaman M, Ioka-Nakamichi T, Inamoto E, Toyonaga H, Mori H. 2005 Complete set of ORF clones of *Escherichia coli* ASKA library (a complete set of *E. coli* K-12 ORF archive): unique resources for biological research. DNA Res. **12**, 291-299. (10.1093/dnares/dsi012) 16769691

[29] Bradford MM. 1976 A rapid and sensitive method for the quantitation of microgram quantities of protein utilizing the principle of protein-dye binding. Anal. Biochem. **72**, 248-254. (10.1006/abio.1976.9999) 942051

[30] Friedman M. 2004 Applications of the ninhydrin reaction for analysis of amino acids, peptides, and proteins to agricultural and biomedical sciences. J. Agr. Food Chem. **52**, 385-406. (10.1021/jf030490p) 14759124

[31] Ellman GL. 1959 Tissue sulfhydryl groups. Arch. Biochem. Biophys. **82**, 70-77. (10.1016/0003-9861(59)90090-6) 13650640

[32] Graf PCF, Martinez-Yamout M, VanHaerents S, Lilie H, Dyson HJ, Jakob U. 2004 Activation of the redox-regulated chaperone Hsp33 by domain unfolding. J. Biol. Chem. **279**, 20 529-20 538. (10.1074/jbc.m401764200) 15023991

[33] Levine RL, Garland D, Oliver CN, Amici A, Climent I, Lenz AG, Ahn BW, Shaltiel S, Stadtman ER. 1990 Determination of carbonyl content in oxidatively modified proteins. Methods Enzymol. **186**, 464-478. (10.1016/0076-6879(90)86141-h) 1978225

[34] Macnair MR, Smirnoff N. 1999 Use of zincon to study uptake and accumulation of zinc by zinc tolerant and hyperaccumulating plants. Commun. Soil Sci. Plan. **30**, 1127-1136. (10.1080/00103629909370273)

[35] Nishikimi M, Appaji N, Yagi K. 1972 The occurrence of superoxide anion in the reaction of reduced phenazine methosulfate and molecular oxygen. Biochem. Biophys. Res. Commun. **46**, 849-854. (10.1016/s0006-291x(72)80218-3) 4400444

[36] Ponti V, Dianzani MU, Cheeseman K, Slater TF. 1978 Studies on the reduction of nitroblue tetrazolium chloride mediated through the action of NADH and phenazine methosulphate. Chem. Biol. Interact. **23**, 281-291. (10.1016/0009-2797(78)90090-x) 214250

[37] Uppu RM, Pryor WA. 1996 Synthesis of peroxynitrite in a two-phase system using isoamyl nitrite and hydrogen peroxide. Anal. Biochem. **236**, 242-249. (10.1006/abio.1996.0162) 8660500

[38] Lamberts JJM, Neckers DC. 1985 Rose Bengal derivatives as singlet oxygen sensitizers. Tetrahedron **41**, 2183-2190. (10.1016/s0040-4020(01)96591-3)

[39] Kochevar IE, Redmond RW. 2000 Photosensitized production of singlet oxygen. Methods Enzymol. **319**, 20-28. (10.1016/s0076-6879(00)19004-4) 10907495

[40] Goldstein S, Meyerstein D, Czapski G. 1993 The Fenton reagents. Free Radical Biol. Med. **15**, 435-445. (10.1016/0891-5849(93)90043-t) 8225025

[41] Qu X, Kirschenbaum LJ, Borish ET. 2000 Hydroxyterephthalate as a fluorescent probe for hydroxyl radicals: application to hair melanin. Photochem. Photobiol. **71**, 307-313. (10.1562/0031-8655(2000)0710307haafpf2.0.co2) 10732448

[42] Brauner A, Fridman O, Gefen O, Balaban NQ. 2016 Distinguishing between resistance, tolerance and persistence to antibiotic treatment. Nat. Rev. Microbiol. **14**, 320-330. (10.1038/nrmicro.2016.34) 27080241

[43] Krewing M, Jarzina F, Dirks T, Schubert B, Benedikt J, Lackmann J-W, Bandow JE. 2019 Plasma-sensitive *Escherichia coli* mutants reveal plasma resistance mechanisms. J. R. Soc. Interface **16**, 20180846. (10.1098/rsif.2018.0846) 30913981 PMC6451402

[44] Bernhardt T, Semmler ML, Schäfer M, Bekeschus S, Emmert S, Boeckmann L. 2019 Plasma medicine: applications of cold atmospheric pressure plasma in dermatology. Oxid. Med. Cell Longev. **2019**, 3873928. (10.1155/2019/3873928) 31565150 PMC6745145

[45] Baba T et al. 2006 Construction of *Escherichia coli* K-12 in-frame, single-gene knockout mutants: the Keio collection. Mol. Syst. Biol. **2**, 473-511. (10.1038/msb4100050) PMC1681482 16738554

[46] Dezest M et al. 2017 Oxidative modification and electrochemical inactivation of *Escherichia coli* upon cold atmospheric pressure plasma exposure. PLoS ONE **12**, e0173618. (10.1371/journal.pone.0173618) 28358809 PMC5373509

[47] Guo L et al. 2018 Cold atmospheric-pressure plasma induces DNA–protein crosslinks through protein oxidation. Free Radical Res. **52**, 783-798. (10.1080/10715762.2018.1471476) 29722278

[48] Dalle-Donne I, Aldini G, Carini M, Colombo R, Rossi R, Milzani A. 2006 Protein carbonylation, cellular dysfunction, and disease progression. J. Cell. Mol. Med. **10**, 389-406. (10.1111/j.1582-4934.2006.tb00407.x) 16796807 PMC3933129

[49] Faulkner KM, Liochev SI, Fridovich I. 1994 Stable Mn(III) porphyrins mimic superoxide dismutase *in vitro* and substitute for it *in vivo*. J. Biol. Chem. **269**, 23 471-23 476. (10.1016/s0021-9258(17)31540-5) 8089112

[50] Egorov SY, Kurella EG, Boldyrev AA, Krasnovsky AA. 1997 Quenching of singlet molecular oxygen by carnosine and related antioxidants. Monitoring 1270-nm phosphorescence in aqueous media. IUBMB Life **41**, 687-694. (10.1080/15216549700201731) 9111930

[51] Lackmann J-W, Bandow JE. 2014 Inactivation of microbes and macromolecules by atmospheric-pressure plasma jets. Appl. Microbiol. Biotechnol. **98**, 6205-6213. (10.1007/s00253-014-5781-9) 24841116

[52] Kuchenbecker M, Bibinov N, Kaemlimg A, Wandke D, Awakowicz P, Viöl W. 2009 Characterization of DBD plasma source for biomedical applications. J. Phys. D: Appl. Phys. **42**, 045212. (10.1088/0022-3727/42/4/045212)

[53] Liochev SI, Fridovich I. 1994 The role of O_2_^−^ in the production of HO^•^: *in vitro* and *in vivo*. Free Radical Biol. Med. **16**, 29-33. (10.1016/0891-5849(94)90239-9) 8299992

[54] Beckman JS, Koppenol WH. 1996 Nitric oxide, superoxide, and peroxynitrite: the good, the bad, and ugly. Am. J. Physiol. Cell Physiol. **271**, 1424-1437. (10.1152/ajpcell.1996.271.5.c1424) 8944624

[55] Haber F, Weiss J. 1932 Über die Katalyse des Hydroperoxydes. Naturwissenschaften **20**, 948-950. (10.1007/bf01504715)

[56] Fridovich I. 1978 The biology of oxygen radicals. Science **201**, 875-880. (10.1126/science.210504) 210504

[57] Lebrun V, Tron A, Scarpantonio L, Lebrun C, Ravanat J, Latour J, McClenaghan ND, Sénèque O. 2014 Efficient oxidation and destabilization of Zn(Cys)_4_ zinc fingers by singlet oxygen. Angew. Chem. Int. Ed. Engl. **53**, 9365-9368. (10.1002/anie.201405333) 25044814

[58] Dirks T, Krewing M, Vogel K, Bandow JE. 2023 The cold atmospheric pressure plasma-generated species superoxide, singlet oxygen and atomic oxygen activate the molecular chaperone Hsp33. Figshare. (10.6084/m9.figshare.c.6875428) PMC10598452 37876273

